# Exploring the Molecular Composition of Dissolved Organic Matter and Its Connection to Microbial Communities in Industrial-Scale Anaerobic Digestion of Chicken Manure

**DOI:** 10.3390/toxics13010049

**Published:** 2025-01-10

**Authors:** Juan Hu, Yurui Zeng, Aibin Hu, Xiaofeng Wang

**Affiliations:** 1 Collaborative Innovation Center for Emissions Trading System Co-Constructed by the Province and Ministry, Wuhan 430205, China; hujuan8108@126.com; 2 School of Discipline Inspection and Supervision, Huanggang Normal University, Huanggang 438000, China; 3 Hubei Key Laboratory of Economic Forest Germplasm Improvement and Resources Comprehensive Utilization, Huanggang Normal University, Huanggang 438000, China; zyrzengyurui@126.com (Y.Z.); huabinhx@126.com (A.H.); 4 School of Computer Science and Hubei Key Laboratory of Intelligent Geo-Information Processing, China University of Geosciences, Wuhan 430078, China

**Keywords:** anaerobic digestion, chicken manure, dissolved organic matter, molecular characterization, microbial community structure

## Abstract

Anaerobic digestion (AD) technology offers significant advantages in addressing environmental issues arising from the intensification of livestock production since it enables waste reduction and energy recovery. However, the molecular composition of dissolved organic matter (DOM) and its linkages to microbial biodiversity during the industrial-scale AD process of chicken manure (CM) remains unclear. In this study, the chemical structure of CM digestate-derived DOM was characterized by using multi-spectroscopic techniques and ultrahigh-resolution mass spectrometry, and the microbial composition was detected by using 16S rRNA gene sequencing. The results revealed that the DOM contained abundant free amino acids and protein-like compounds but fewer humic-like substances, identified as lignin/carboxylate-rich alicyclic molecules, lipids, and proteins/aliphatic compounds featuring enriched S_5–6_O_1_ and N_1–5_O_X_ fragments. In addition, the 16S rRNA results revealed microorganisms that were centered on metabolic function in the production of volatile fatty acids, H_2_S/CH_4_, and the hydrolysis reaction in the AD process. Free amino acids and protein-like compounds were mainly associated with hydrolysis reactions and H_2_S production functional microorganisms. Lignin/carboxylate-rich alicyclic molecules were linked to microorganisms possessing hydrolysis reactions and, indirectly, CH_4_ production. This study elucidates the linkage with the microbial and molecular composition of DOM, establishing a theoretical foundation for employing AD in the disposal of CM.

## 1. Introduction

The large-scale development of the livestock and poultry industry has resulted in the rapidly increasing production of organic solid waste. The annual production of animal manure amounts to 3800 million tons in China, especially chicken manure (CM), which accounts for about 28% of the total amount and displays the fastest-growing resource among these in recent years [1]. However, plenty of recyclable substances and energies contained in organic solid waste have not been effectively used. Compared to other treatment technologies including composting, aerobic treatment, and black soldier fly treatment [2,3], anaerobic digestion (AD) is an environmentally sustainable waste treatment technology that concurrently reduces pollutants and recycles value-added products (i.e., energy, fatty acids, and nutrient recovery) [4,5]. Moreover, chicken manure featuring a high-solids character has advantages for AD treatment, generating a final byproduct, including methane production and organic fertilizer (digestate and liquid fractions of digestates) [6]. Therefore, a comprehensive characterization of the AD liquid from full-scale anaerobic digestion (AD) of chicken manure is essential for the safe use of manure as an efficient fertilizer in agricultural fields.

Previous studies have concentrated on enhancing the methane production from AD of chicken manure by improving various methods, including hydrothermal pretreatment, enchantment of microbial viability, in situ ammonia stripping, and the co-digestion process [7,8]. The increase in methane production has been attributed to enhanced metagenomic analyses, improving dissolved organic matter (DOM) biodegradation and subsequently enhancing methane production. Since DOM is a primary component of anaerobic digestion liquid, researchers have focused on using multispectral analysis in combination with nuclear magnetic resonance to analyze the physicochemical properties of AD liquid, determining the chemical composition involved in the function group information of DOM [9,10]. Recently, with the advancement of ultra-high-resolution mass spectrometry, it has found increasing application in the field of wastewater and sludge treatment [11,12]. Fourier transform ion cyclotron resonance mass spectrometry (FT-ICR MS) enables precise determination of the molecular formula of DOM, making it better suited for the characterization of natural DOM [13]. However, the composition of DOM at the molecular level remains unclear for an industrial-scale AD of chicken manure.

Moreover, the anaerobic digestion process is mediated by various types of microorganisms; thus, microbial activity affects the chemical transformation of DOM, thereby influencing the characteristics of anaerobic digestion liquid. According to previous literature, statistical analysis results have uncovered that both aceticlastic (i.e., syntrophic acetate oxidation bacteria and Bacteroidetes phyla) and hydrogenotrophic methanogenesis (i.e., *Methanosaeta* sp. and *Methanobacterium*) are dominant in the microbial community structure of the AD process, and their relative abundance depends on environmental conditions [14]. For example, the microbial network is strongly affected by the organic loading rate, with the increase in which the *Methanosaeta* sp. was gradually replaced by *Methanosarcina* sp. and the acetogenic community structure in the AD [15]. However, the molecular-level transformation mechanism of DOM driven by the microbial community in the AD of chicken manure remains unclear. Some studies have claimed that the enhancement of microbial activity improves substance metabolism levels, benefiting the conversion of protein-like DOM to volatile fatty acids. In turn, the increase in easily biodegradable DOM and protein-like substances greatly improves the substrate’s biodegradability and microbial metabolism capacity, suggesting that strong correlations are found between topological properties of DOM–bacteria associations and protein-like DOM in AD [16]. Hence, the comprehension of this mechanism is highly beneficial for understanding the interactive correlation between the transformation of DOM in the progression of AD.

Considering the investigation of the molecular characters of DOM derived from CM organic matter microbial metabolites and its relationship with methanogenic microbes in a stable operational state is essential, as monitoring these parameters during the anaerobic digestion process facilitates the optimization of CH_4_ production. Therefore, the purposes of this study are to (1) reveal the molecular composition of DOM extracted from industrial-scale anaerobic digestion of chicken manure using FT-ICR MS coupled with multispectral analysis; and (2) shed light on the interactions between the microbial community structure and the molecular composition of DOM using network analysis. This study clarifies the relationship between molecular composition and microbial communities, establishing a theoretical basis for the application of anaerobic digestion in the management of CM.

## 2. Materials and Methods

### 2.1. Sample Sources

The liquid fraction of digestates were obtained from industrial-scale anaerobic digestion of chicken manure from a plant in Xinzhou district, Wuhan, China. The project was designed to process 150 tons of CM per day, resulting in an annual production of 2555 tons of solid organic fertilizer and 8760 tons of liquid digestate per year. The main process flow includes a chicken manure pretreatment unit, a high-temperature hydrolysis acidification unit, medium temperature anaerobic digestion, a deep anaerobic reactor, and a solid–liquid separation equipment discharge unit. The working temperature of the anaerobic digestion process was 35 °C, and the hydraulic retention time was 15 d. The volume of the monomer was 2000 m^3^, and the size of the monomer was φ14.5 m × 13 m. The non-standard side-entry agitator was used for mixing. The CH_4_ yield was about 1.9 t/d. The generated biogas was used for electricity generation, while the digestate and bio-slurry were utilized as fertilizers. DOM was extracted from digestion liquid according to previous references [11]. Briefly, after transporting the digestion liquid stored in dry ice to the laboratory, the sample was transferred to a 50-mL tube (Biosharp, Labgic Technology Co., Ltd., Beijing, China) and centrifuged at 4000 g/min for 30 min using a L535R centrifuge (Xiangyi, Xiangtan, China), followed by the collection of the supernatant. After that, DOM samples were acquired by filtering the supernatants via a 0.45-µm glass fiber membrane (Xingya, Shanghai, China). Finally, the prepared samples were stored at 4 °C prior to characterizing DOMs.

### 2.2. Bulk Measurements Analysis of DOMs

The fundamental physical and chemical properties of organic matters include pH, electrical conductivity (EC), dissolved organic carbon (DOC), and the specific UV absorbance at 254 nm (SUVA_254_). The value of DOC was detected by catalytic combustion and the non-dispersive infrared detection method with a total organic carbon analyzer (Teledyne Tekmar, Torch, USA). Briefly, after the anaerobic digestion liquid was passed through a 0.45-µm glass fiber membrane, the filtered solution was placed in a 40-mL injection bottle and sealed with a sealing film. Then, the sample was analyzed using a total organic carbon analyzer, and the contents of total carbon and total inorganic carbon were obtained. The value of total carbon and total inorganic carbon were subtracted according to a standard curve; that is, the value of total organic carbon was obtained. The pH/EC and SUVA_254_ were analyzed using a pH meter (Mettler-Toledo, Switzerland) and an ultraviolet-visible (UV-vis) spectrophotometer (Shimadzu UV-1900, Shimadzu, Kyoto, Japan), respectively. The SUVA_254_ value was calculated to evaluate the aromatic structure of DOMs, using the following equation: SUVA_254_ = UV_254_ × 100/DOC, where UV_254_ is the UV-vis absorbance at a wavelength of 254 nm and DOC represents the DOM concentration. Fourier-transformed infrared spectroscopy (FT-IR, Nicolet iS10, Thermo Fisher Scientific, USA) was used to analyze the functional groups and measured by the KBr pellet technique in the wavenumber range of 400–4000 cm^−1^. The concentrations of total phosphorus (TP), total nitrogen (TN), NH_4_^+^-N, and PO_4_^3−^ were determined according to EPA Methods [17]. The humic acid, protein, and free amino acid concentrations in DOMs were determined following previous references [18,19] A three-dimensional excitation–emission matrix (3D-EEM) was obtained using a fluorescence spectrophotometer (F-4500, Hitachi, Japan). The emission spectra were scanned from 220 to 600 nm in 2-nm steps, and the excitation spectra were recorded from 200 to 500 nm in 5-nm increments. The free amino acid content was detected by using reverse-phase high-performance liquid chromatography. The content of crude proteins was quantified based on the assessment value of organic nitrogen, with a nitrogen-to-protein conversion factor of 6.25 [19]. Humic acid concentration was determined by using the determination of humic acids in water-soluble fertilizers according to the NY-T 1971-2010 standard [20]. All tests were carried out at least in triplicate.

### 2.3. Molecular Compositions of DOM Analyzed by FT-ICR MS

The DOM samples were purified by the solid phase extraction method using Agilent Bond Elut-PPL cartridges (500 mg per 6 mL), as suggested in our previous work [21]. In summary, the cartridges were sequentially rinsed with 12 mL of methanol (LC-MS grade) and acidified ultra-pure water (pH = 2), which was diluted with hydrochloric acid before being used. After that, about 50 mL DOM solution at pH = 2 was filtered through the cartridges using gravity. The cartridges were initially rinsed with 12 mL of acidified ultra-pure water (pH = 2) to eliminate impurities, dried using a stream of nitrogen, and promptly extracted with 6 mL of methanol. The residual eluted samples were blow-dried with nitrogen and then reconstituted in 1 mL of a methanol/water mixture (v/v = 1) for analysis using FT-ICR MS. The resolution of FT-ICR MS data referred to prior studies, including the external and internal calibration of mass spectra, extraction of the molecular ion peak, identification of all possible molecular formulae, and matching of molecular formulae. Subsequently, the obtained chemical molecular formula (i.e., C_x_H_i_O_j_N_k_S_l_), was utilized for calculating fundamental molecular parameters, such as the calculation of the double-bond equivalence (DBE), which was was performed as follows: DBE = (2C + 2 − H + N)/2. The modified aromaticity index (AI_mod_) was determined using the following equation: AI_mod_ = (1 + C − 0.5O−S − 0.5 H)/(C − 0.5C – S − N) [22].

### 2.4. Microbial Community Structure Analyzed by High-Throughput Sequencing

Microbial community analysis samples were obtained from an industrial-scale AD of chicken manure, and these were then stored at −20 °C until use. The DNA extracted from the AD sample was extracted using a MagicPure^®^ Soil Genomic DNA Kit (Findrop Biosafety Technology Co., Ltd., Guangzhou, China). Thereafter, the V4 region was amplified by PCR using primers 338F (ACTCCTACGGGAGGCAGCA) and 806R (GGACTACHVGGGTWTCTAAT) [23]. The 16S rRNA gene was sequenced on the Illumina Hiseq 2500 platform (Guangdong Magigene Biotechnology Co., Ltd., Guangzhou, China) [24]. Sequencing libraries were prepared with the NEBNext^®^ Ultra™ II DNA Library Prep Kit for Illumina^®^ (New England Biolabs, Ipswich, MA, USA) according to the manufacturer’s instructions, incorporating index codes. The library quality was evaluated using a Qubit^®^ 2.0 Fluorometer (Thermo Fisher Scientific, Waltham, MA, USA). Thereafter, the libraries were sequenced on the Illumina Nova6000 platform, producing 250 bp paired-end reads. The average quality value and length of the processed data were greater than 20 and longer than 100 bp, and the data were clustered into amplicon sequence variants at a 100% similarity level using QIIME2 (v2020.11.0). Then, each ASV was assigned to a taxonomic level through the Silva 138 database using qiime feature-classifier classify-sklearn. The alpha diversity of each compost sample was determined by using search v10.0.240 software.

### 2.5. Data Processing

FRI analysis was carried out by MATLAB code in-house via MATLAB (2021b, MathWorks, Natick, MA, MA) to determine the fluorescent components of CM digestate-derived DOM [11,25]. In brief, before conducting the FRI analysis, correction procedures including inner filter correction, blank subtraction, and normalization of Raman scattering units were performed. Then, according to the already defined five regions of EEM, the normalized excitation–emission area volumes of five regions were calculated according to Equations (1) and (2), and subsequently, the percent fluorescence response was obtained by the calculation of Equation (3).

Φi,n = MFiΦi = MFi∑e_x_∑e_m_I(λ_ex_λ_em_)Δλ_ex_Δλ_em_ (1)

ΦT,n = ∑Φi,n(2)

Pi,n = Φi,n/ΦT,n × 100%(3)

where Δλ_ex_ is the excitation wavelength interval (taken as 5 nm), Δλ_em_ is the emission wavelength interval (taken as 5 nm), and I (λ_ex_λ_em_) is the fluorescence intensity (au) at each excitation-emission wavelength pair. MFi is a multiplication factor, equal to the inverse of the fractional projected excitation–emission area.

The relationship between the molecular structures of DOM, including humic acids, FRI result, molecular composition, and the metabolic reaction of microorganisms was determined, and then, the related microbial species that possess metabolic reactions were identified as the most abundant microbial species. All data were processed using Origin 2021 software.

## 3. Results and Discussion

### 3.1. Bulk Properties of Characteristics of CM Digestate-Derived DOM

**Figure 1:**
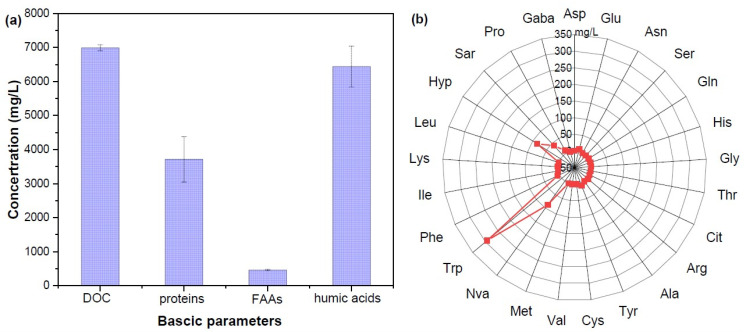
Basic characteristics (**a**) and 17 kinds of free amino acids (**b**) in CM digestate-derived DOM.

DOM composition analysis in CM digestates revealed that it had a high concentration of dissolved organic carbon (DOC) (6990.83 mg/L), total nitrogen (TN) (5.1 g/L), total potassium (TK) (3.37 mg/L), ortho-phosphate (PO_4_^3−^) (26.1 mg/L), and ammonium ion (NH_4_^+^) (4506 mg/L) (Table 1 and Figure 1). The nutrient content (PO_4_^3−^, K^+^, and NH_4_^+^) is beneficial for plant growth, but higher concentrations of NH_4_^+^ and high salt conditions impede the metabolic processes of bacteria and plants [26]. A higher NH_4_^+^ content can significantly inhibit anaerobic digestion of CM for methane production. In addition, DOM was found to contain more kinds of bio-stimulants that promote plant growth, such as proteins, free amino acids (FAA), and humic acid (Figure 1a). The protein content of CM digestate DOM was 3712.31 mg/L, which may be ascribed to the hydrolyzed protein products, including soluble small proteins, polypeptides, and amino acids, since CM was enriched in more protein-like compounds [27]. Further study found that the total concentration of 17 kinds of FAA was 545.263 mg/L. The top six most abundant FAA classes in CM digestate-derived DOM were tryptophan (292.98 mg/L), norvaline (87.80 mg/L), hydroxyproline (83.74 mg/L), sarcosine (41.00 mg/L), proline (8.67 mg/L), and tyrosine (8.17 mg/L) (Figure 1b). It has been proven that tryptophan and its metabolites can be used as an effective indicator of pollution and biological metabolites since microbes are able to convert tryptophan into various molecules, such as indole and its derivatives [28]. Moreover, the humic acid content of CM digestate DOM was detected as 6.44 g/L, which was dominantly composed of phenols, ketones, and long-chain fatty acids [29]. A previous study has indicated that humic acid contained in CM can inhibit the conversion efficiency of methane, and its formation and transformation are controlled by the dynamic change of amino acids, polyphenols, and quinone compounds according to the polyphenol humification pathway [30].

**Figure 2:**
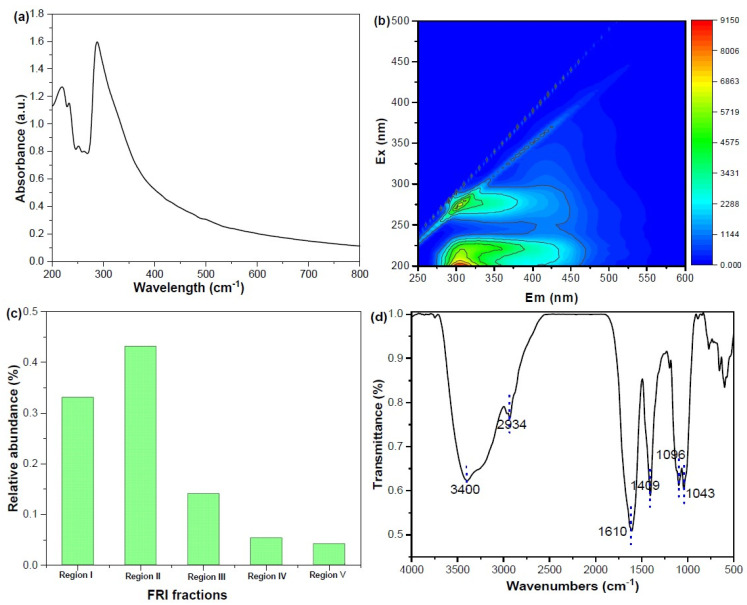
UV–vis spectrum (**a**), fluorescent characteristics (**b**), and FRI analysis derived from the EEM spectrum (**c**), and FTIR spectrum of CM digestate-derived DOM (**d**).

The chemical structures of CM digestate DOM were further assessed by multiple spectroscopic analysis, including UV–vis, EEM, and FTIR spectroscopy (Figure 2). The value of SUVA_254_ determined the content of unsaturated C=C and aromatic compounds, indicating the aromatic property of organic matter [31]. It was found that the value of SUVA_254_ in CM digestate-derived DOM was 0.24, lower than that of other source digestates (such as corn stover and sludge). This might be due to higher levels of protein compounds in CM that could not provide sufficient precursors for the synthesis of humic-like substances [32]. It indicates that CM digestate DOM contains a relatively higher content of protein-like components and lower content of aromatic compounds. This finding was further confirmed by the EEM and FRI analysis. FRI is a quantitative technique that integrates volumes beneath various excitation and emission regions in EEM spectra, which enables the quantitative analysis of EEM spectra and facilitates the determination of the heterogeneity of DOM. EEM spectroscopy of CM digestate DOM showed that a peak appeared at an Ex/Em of 200–250/380–500 nm, which could be categorized as fulvic-like materials (belonging to Region III). Two peaks were located at an Ex/Em of 250–280/200–380 nm (belonging to Region IV) and 200–250/200–330 nm (belonging to Region I). These Ex/Em values corresponded to tryptophan-like and tyrosine-like materials according to previous references [25,33]. The relative abundance of fulvic-like materials (III) and humic acid-like materials (V) were 14.11% and 4.2%, respectively. Region II located at an Ex/Em of 200–250/330–380 nm was related to tryptophan-like material [33]. Quantitative FRI analysis obtained from EEM spectroscopy of DOM showed that protein-like materials (the tryptophan-like and tyrosine-like materials) are the most dominant fluorescent components, accounting for 80%, with tryptophan-like material of 40%, in digestate DOM. In addition, the FT-IR spectrum was used to depict the functional groups of digestate DOM. It was found that five characteristic absorption bands had several peaks at 3400, 2934, 1610, 1409, and 1096 cm^−1^, indicating O-H/N-H groups, aliphatic C-H stretching, C=O in amides, carboxylic acids, and C-O-C groups, respectively [34]. The results implied that CM digestates had typical protein-like substances (3300, 1610, and 1409 cm^−1^), humic-like substances (3300, 2934, and 1096 cm^−1^), and polysaccharide-like substances (3300, 2934, and 1096 cm^−1^). In summary, the above-mentioned results showed that these compounds in CM digestate-derived DOM contained more free amino acids and protein-like materials and less humic-like substance materials.

### 3.2. Molecular Composition of CM Digestate-Derived DOM by FT-ICR MS

**Figure 3:**
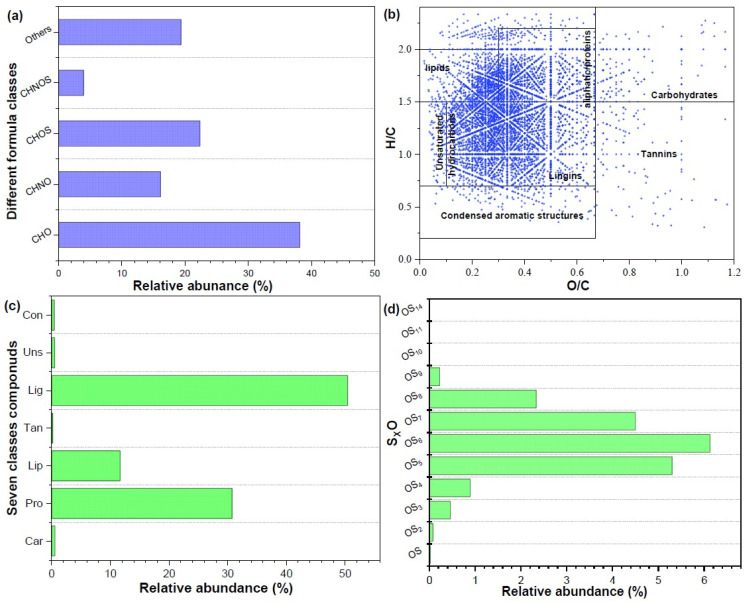
The relative abundance of molecular compositions based on different elemental composition (**a**), Van Krevelen diagrams of CM digestate-derived DOM (**b**), percentage of formulas within the seven groups in DOM (**c**), and the distribution of S_x_O_1_ compounds in DOM (**d**).

The further molecular composition of CM digestate DOM revealed by FT-ICR MS showed that heteroatom-containing compounds, including CHON, CHOS, CHONS, and p-containing compounds, accounted for more than 60%, especially CHOS (22.38%) and p-containing compounds (19.37%) (Figure 3a). The p-containing compounds in DOM were attributed to microbial metabolism or cell lysis, and CHOS/ONS compounds belonged to the hydrolysis or micro-biological degradation products of proteins in CM digestate-derived DOM [35]. This result was further confirmed by the distribution patterns of S_x_O_1,_ O_x_ and N_1–5_O_X_ class compounds. It was found that S_5–6_O_1,_ and N_1–5_O_X_ dominated the in heteroatom-containing compounds of DOM (Figure 3d). Meanwhile, the dominant oxygen-containing molecule was O_4–6_ class compounds. The chemical composition of CM digestate DOM was obviously distinguished from other organic solid waste source-derived DOMs, such as sewage sludge/kitchen waste digestate-derived DOM, which contained about 56% (30%) CHON compounds [11,36]. The observed phenomenon may be due to the high content of proteins (including s-containing protein) and enteric microorganisms present in CM digestates [37]. Additionally, according to the Van Krevelen diagram constructed by H/C and O/C ratios, CM digestate-derived DOM were divided into seven classes including lipids (H/C = 1.5–2.0, O/C = 0–0.3), proteins/aliphatic compound (H/C = 1.5–2.2, O/C = 0.3–0.67), lignin/carboxylic rich alicyclic molecules (CRAM) (H/C = 0.7–1.5, O/C = 0.1–0.67), carbohydrates (H/C = 1.5–2.4, O/C = 0.67–1.2), unsaturated hydrocarbons (H/C = 0.7–1.5, O/C = 0–0.1), aromatic structures (H/C = 0.2–0.7, O/C = 0–0.67), and tannin (H/C = 0.6–1.5, O/C = 0.67–1.0). Figure 3b reveals that these points in the Van Krevelen diagram from CM digestate DOM were primarily focused on the lipids, aliphatic/proteins, and lignin/CRAM regions. Further semi-quantitative analysis showed that the top three substances were lignin/CRAM (50.48%), proteins/aliphatic compounds (30.76%), and lipids (11.67%) (Figure 3c). Proteins in CM digestate DOM were attributed to the amino acid, polypeptide, and small molecule proteins, and lipids were identified as short/long-chain fatty acids. These were identified as the most important fraction for fertilization. Lignin/CRAM from CM digestate DOM was possibly ascribed to refractory organic matter, including humic acid/fulvic-like substances and refractory microbial products with structural similarities to sterols and terpenoids [38,39]. A similar result has been documented in other anaerobic digestion processes for solid organic waste [40]. In summary, CM digestate DOM comprised lignin/CRAM, lipids, and proteins/aliphatic compounds with enriched S_5–6_O_1,_ and N_1–5_O_X_ fragments.

### 3.3. Microbial Structure Composition Analysis of CM Digestate-Derived DOM

**Figure 4:**
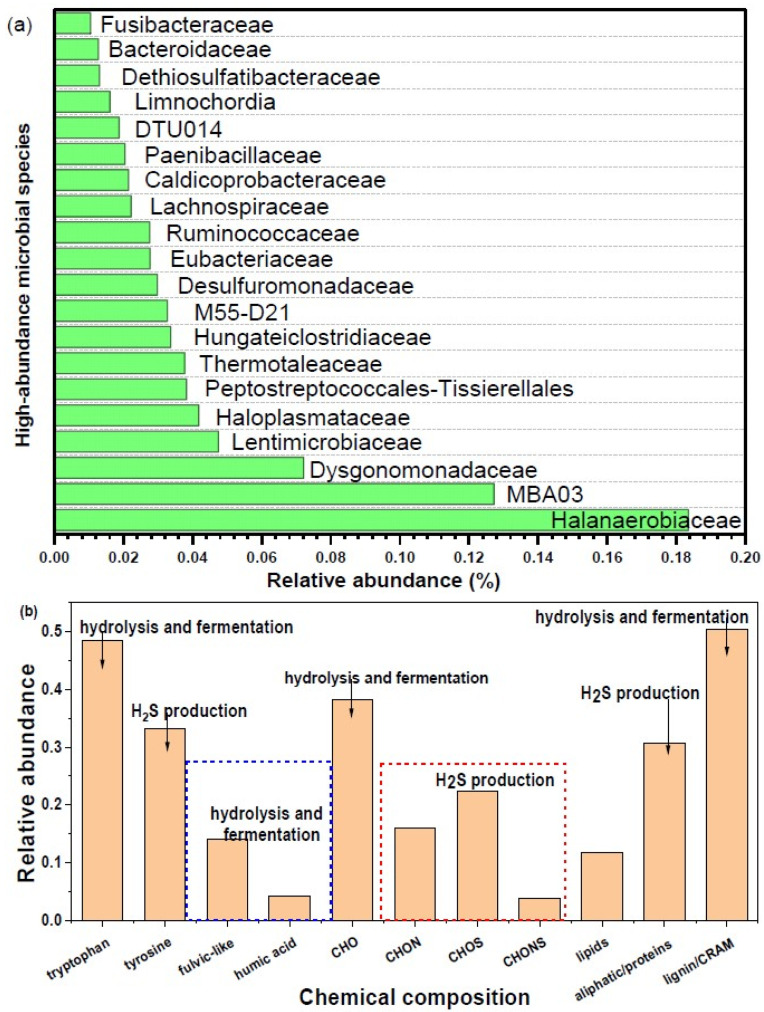
Top 20 most abundant microorganisms of all bacterial composition at the family class level (**a**), and the relationship between the chemical composition of DOM and microbial structure composition (**b**).

The major microbial structure composition is illustrated in Figure 4a. The top twenty most abundant families comprised gastrointestinal tract bacterial communities of chickens, including Peptostreptococcales-Tissierellales, Thermotaleaceae, and Paenibacillaceae, and microorganisms involved in anaerobic digestion, including Halanaerobiaceae, Dysgonomonadaceae, MBA03, Dysgonomonadaceae, Lachnospiraceae, Dethiosulfatibacteraceae, and Fusibacteraceae. The function of microorganisms involved in the anaerobic digestion process were centered on volatile fatty acid production (29.5% in abundance), synergistic effects for CH_4_ production (25.6% in abundance) [41], hydrolysis and fermentation reactions (11.3% in abundance) [42], and H_2_S production (7.6% in abundance). Generally speaking, the dominant anaerobic digestion-related microorganisms were Halanaerobiaceae (18.4% in abundance), MBA03 (12.7% in abundance), and Dysgonomonadaceae (7.2% in abundance). The Halanaerobiaceae bacteria, with high salt tolerances, similar to homoacetogens, participate in acetic acid fermentation metabolism [43]. The MBA03 family is widely distributed in the anaerobic digestion process and regarded as a potential key player in anaerobic digestion, contributing to methane production together with hydrogenotrophic archaea [44]. The Dysgonomonadaceae and Synergistaceae families of acetate-oxidizing bacteria were responsible for the production of methane [45]. Additionally, there were some special microbial structure compositions for the CM anaerobic digestion process. For example, Dethiosulfatibacteraceae bacteria degrade amino acids and proteins, leading to the production of non-sulfate H_2_S [46], similar to the M55-D21 and Desulfuromonadaceae families [47]. Finally, the relationship between the abundant bacteria composition and the chemical composition of CM digestate-derived DOM to identify the key molecules and microorganisms(Figure 4b). It was found that the highest percentage of microorganisms possess volatile fatty acid production functions, participating in converting the primary hydrolysis products into volatile fatty acids, carbon dioxide, and small organic matters. Consequently, plenty of lipid-like compounds are produced during the CM anaerobic digestion process. Notably, microorganisms were only involved in the synergistic effect for CH_4_ production, and no microorganisms involved in the direct generation of CH_4_ production were identified by the 16S test of CM digestates. This occurs because methane production is biologically dominated by methanogenic archaea, which are not detected without the addition of suitable primers for 16S analysis of CM digestates. Additionally, microorganisms with a H_2_S production function and a high relative abundance were related to the transformation of protein degradation to amino acid, according to the presence of high percentages of proteins and amino acids in CM digestate-derived DOM. Last but not least, the generation of humic acid-like materials was involved in the decomposition and repolymerization reaction, which was possibly associated with those microorganisms participating in hydrolysis of proteins, polysaccharides, and lipids, reducing microorganisms and synergistic effects for CH_4_ production during the anaerobic digestion process of CM [48]. The findings reveal the key microbial composition of the translation of DOM, deepen our understanding of the link between the molecular composition and microbial composition, and provide a theoretical basis for using the anaerobic digestion process for disposal of CM.

## 4. Conclusions

Investigating the molecular characters of DOM derived from CM organic matter microbial transformation products and its relationship with methanogenic microbes in a stable operational state is essential, as monitoring these parameters during the anaerobic digestion process facilitates the optimization of CH_4_ production. However, this related work remains poorly understood. Our findings indicate that the CM digestate-derived DOM contained more free amino acids and protein-like compounds but less humic-like substance materials. These were identified as lignin/CRAM, lipids, and proteins/aliphatic compounds with enriched S_5–6_O_1_, and N_1–5_O_X_ fractions by FT-ICR MS. In addition, high-throughput sequencing revealed that the function of microorganisms was centered on volatile fatty acid production, synergistic effects for CH_4_ production, hydrolysis and fermentation reactions, and H_2_S production. The linkage of the molecular composition of DOM and microbial biodiversity revealed that free amino acids and protein-like compounds were closely related to these microorganisms functioning in hydrolysis reactions and H_2_S production. These were correlated with the chemical composition of CM digestate-derived DOM, especially free amino acids and protein-like compounds. Further work should be focused on network analysis between the molecule composition and the metagenomics of microorganisms to identify the key molecules and microorganisms. This study elucidates the relationship between molecular composition and microbial composition, providing a theoretical foundation for employing anaerobic digestion in the disposal of CM.

## Figures and Tables

**Table 1:** Basic characteristics of CM digestate-derived DOM.

Indicators	TN	PO_4_^3−^	TK	NH_4_^+^-N	pH	EC
Value	5100 ± 100	26.1 ± 10	3366.7 ± 57.7	4506 ± 12.8	8.14 ± 0.01	32.2 ± 0.3

Note: the unit of TN, PO_4_^3−^, TK, and NH_4_^+^-N was mg/L, and of EC was ms/cm^3^.

## Data Availability

The original contributions presented in this study are included in the article. Further inquiries can be directed to the corresponding author.
